# Whose fears count? Legitimacy, trust and viral outbreak responses after COVID-19

**DOI:** 10.1371/journal.pmed.1005184

**Published:** 2026-07-22

**Authors:** Rebecca F. Grais

**Affiliations:** Pasteur Network, Paris, France

## Abstract

In this Editorial, Rebecca Grais outlines how COVID-19 transformed viral outbreak responses from a predominantly public health challenge into a political and security concern, highlighting the challenges of ensuring that competing perceptions of risk are managed in ways that are perceived as legitimate, fair, and inclusive.

## Teaser

COVID-19 transformed viral outbreak response from a predominantly public health challenge into a political and security concern. Recent responses to Ebola, mpox, and other emerging infections suggest that preparedness is increasingly shaped by whose fears are prioritized in decision-making. The challenge is ensuring that competing perceptions of risk are managed in ways that are perceived as legitimate, fair, and inclusive.

Historically, viral outbreak responses have focused primarily on populations directly affected by disease. During Ebola outbreaks in West Africa and the Democratic Republic of the Congo (DRC), principle concerns centered on transmission, access to care, frontline worker safety, and the social consequences of control measures [1–4]. Community engagement became increasingly recognized as essential because public cooperation influenced whether interventions succeeded. Debates about trust, resistance, and misinformation emerged largely in relation to populations experiencing the outbreak itself. Before COVID-19, epidemic preparedness was largely understood as a public health challenge requiring surveillance, diagnostics, vaccines, treatment, and community engagement. After COVID-19, however, outbreaks increasingly became matters of national security, economic stability, border control, and domestic political risk. Public health responses have always involved balancing competing interests and risks. Understanding this shift is essential because preparedness now requires institutions to govern competing perceptions of risk—not simply infectious threats themselves.

The COVID-19 pandemic demonstrated how rapidly an infectious disease threat could disrupt economies, political systems, education, international travel, and global supply chains [5,6]. High-income governments discovered that public perceptions of risk could have consequences extending far beyond health outcomes. Border closures, school closures, lockdowns, travel restrictions, vaccine procurement, and economic recovery became matters of national political importance. Outbreak response became linked not only to health protection but also to political legitimacy, economic resilience, and national security.

As a result, contemporary outbreak responses often involve a broader range of actors than before COVID-19. Public health agencies now often operate directly alongside ministries of finance, interior ministries, security agencies, border authorities, and political leaders. These actors may perceive different risks and prioritize different outcomes. The result is not simply more stakeholders. It is a different hierarchy of concerns. The question is therefore not only whether outbreaks generate fear: It is whose fear matters most when decisions are made.

Yet fear itself is an imperfect indicator of public confidence. During some outbreaks, mistrust manifests not as excessive fear but as its opposite. Communities that distrust governments or health authorities may dismiss official warnings, reject public health advice, or interpret outbreak messaging through political rather than scientific lenses [1–3]. The challenge for preparedness is therefore not simply managing fear but understanding how trust and mistrust shape perceptions of risk in both directions.

The ongoing outbreak of Ebola disease caused by Bundibugyo virus in the DRC and Uganda illustrates this challenge. Declared a Public Health Emergency of International Concern in May 2026, the outbreak is occurring in a context of insecurity, population displacement, cross-border movement, and substantial scientific uncertainty [7,8]. Unlike outbreaks caused by Zaire ebolavirus, Bundibugyo virus disease remains comparatively understudied, with limited evidence regarding vaccines, therapeutics, and clinical management [9]. For communities living through the outbreak, the most immediate concerns are often practical rather than geopolitical. Families may fear separation from relatives admitted to treatment centers and not being able to bury their loved ones. Communities may worry about movement restrictions, disruptions to livelihoods, or access to healthcare. Healthcare workers may fear infection while operating in settings with limited resources and uncertain treatment options.

Governments may perceive different risks. Political leaders may worry about public confidence, economic disruption, or criticism regarding outbreak management. Neighboring countries may focus on cross-border transmission. International actors may focus on preventing regional or global spread. High-income countries may view outbreaks through the lens of importation risk and preparedness.

The international response to mpox illustrates a similar dynamic. Clade I mpox circulated in parts of Central Africa for years with relatively limited international attention. Global concern increased substantially when transmission occurred outside Africa in 2022 and has intensified again with recent outbreaks. In effect, international concern expanded only once the fears of populations outside endemic regions entered political decision-making. These concerns are legitimate. However, the contrast highlights longstanding disparities in attention, surveillance investment, vaccine availability, and political urgency. The differing responses raise important questions regarding which outbreaks generate action and whose risks are perceived as globally relevant.

COVID-19 brought these competing priorities into unusually sharp focus because governments around the world were simultaneously balancing domestic political pressures and global public health responsibilities. For example, vaccine nationalism emerged because governments prioritized protecting their own populations, often at the expense of broader global solidarity [10]. Yet inequity itself was not new. Access to HIV treatment, cholera vaccines, diagnostics, and essential medicines among others have long reflected global disparities in power and resources. COVID-19 did not create these inequities; it made them politically impossible to ignore. It revealed how rapidly governments privilege domestic political legitimacy over global solidarity when faced with uncertainty. Travel restrictions were frequently implemented despite limited evidence of effectiveness, in part because governments faced pressure to demonstrate visible action [6,11]. Decisions were often shaped not only by epidemiological evidence but also by political and societal expectations.

The same tensions can be observed in contemporary responses to Ebola and other emerging pathogens. Discussions regarding patient evacuation, travel restrictions, border controls, and access to investigational countermeasures often reflect competing perceptions of fear and acceptable risk [5,11]. During several Ebola outbreaks, healthcare workers from high-income countries were evacuated for treatment while local healthcare workers remained in resource-constrained settings. Such decisions may be medically justified, but they are also interpreted through the lens of fairness and reciprocity. Experiences of corruption, exclusion, poor service delivery, and political marginalization can also shape how communities interpret public health measures when emergencies occur [1–3]. Where institutions are perceived as corrupt, unresponsive, or inequitable before a crisis, public health authorities may enter outbreaks with a substantial legitimacy deficit already in place.

Public trust therefore remains essential, but the central governance challenge after COVID-19 is how institutions balance competing claims about whose risks deserve priority when evidence is uncertain and resources are limited.

Much of the literature on outbreak response focuses on communication, misinformation, and community engagement [1–4]. These issues remain important. However, they do not fully explain why some response measures are accepted and others are contested. Acceptance often depends less on whether populations agree with decisions than on whether they believe their concerns were considered during decision-making. Research has consistently shown that people are more likely to accept difficult decisions when decision-making processes are perceived as fair, transparent, and inclusive. In outbreak settings characterized by uncertainty, this may be particularly important [12,13]. Acceptance may also depend on reciprocity. During the West African Ebola epidemic, communities often accepted substantial restrictions on movement, burial practices, and social life when these measures were accompanied by tangible support, including healthcare access for non-outbreak-related conditions, food assistance, and protection of livelihoods. Reciprocity transformed restrictive measures from externally imposed obligations into collectively shared responses [14].

Recent public reactions to reports of Hantavirus infections provide another illustration of how outbreak perceptions have changed after COVID-19. Although Hantaviruses are fundamentally different from SARS-CoV-2 and pose limited pandemic risk, media coverage and public discussion rapidly invoked comparisons with COVID-19. Such reactions suggest that many populations continue to interpret emerging infectious disease threats through the lens of recent pandemic experience, creating additional pressures on governments and public health authorities to respond to fears that may not always align with epidemiological risk.

Preparedness frameworks have been slow to recognize this shift. Most preparedness metrics focus on laboratory capacity, surveillance systems, diagnostics, vaccines, response timelines, and stockpiles. These capabilities remain essential. However, they reveal little about how societies manage competing perceptions of risk.

The central challenge facing outbreak preparedness after COVID-19 may therefore be different from the one that existed before the pandemic. The question is no longer simply whether institutions can persuade populations to trust them. Nor is it whether sufficient technical capacity exists to respond to emerging threats. Increasingly, the challenge is whether institutions can govern uncertainty and balance competing fears in ways that are perceived as legitimate and fair. Institutions must develop decision-making processes that visibly balance competing risks, explain trade-offs transparently, and demonstrate that those bearing the greatest burdens have genuine influence over how outbreak policies are designed. Preparedness assessments should therefore evaluate not only laboratories, surveillance systems, and countermeasures, but also whether institutions possess mechanisms for transparent decision-making, meaningful public participation, reciprocal support for populations bearing the greatest burdens, and the legitimacy to balance competing fears during future outbreaks.

## Abbreviation:

DRC: Democratic Republic of the Congo
